# BMC Ear, Nose and Throat Disorders reviewer acknowledgement 2014

**DOI:** 10.1186/s12901-015-0014-0

**Published:** 2015-02-10

**Authors:** Magdalena Morawska

**Affiliations:** BioMed Central, Floor 6, 236 Gray’s Inn Road, London, WC1X 8HB UK

## Abstract

The editors of *BMC Ear*, *Nose and Throat Disorders* would like to thank all of our reviewers who have contributed to the journal in Volume 14 (2014).

**Ilona Anderson**

Austria

**Minakshi Bhardwaj**

India

**Marc Braem**

Belgium

**Itzhak Brook**

USA

**José Cameselle-Teijeiro**

Spain

**Peter W. Dettmar**

UK

**Y.P. Peter Di**

USA

**Wouter Dreschler**

Netherlands

**Caterina Finizia**

Sweden

**Elizabeth Fitzpatrick**

Canada

**Noriyuki Fujima**

Japan

**Karyn Galvin**

Australia

**Miguel Goncalves**

UK

**Hasantha Gunasekera**

Australia

**Hervé Haas**

France

**Stefan Hegemann**

Switzerland

**Sebastian Hoth**

Germany

**Hiroaki Ichijo**

Japan

**Takao Imai**

Japan

**Fikret Kasapoglu**

Turkey

**Tejs Klug**

Denmark

**Masahiro Komori**

Japan

**Kenji Kondo**

Japan

**Seung-Han Lee**

South Korea

**David J Lim**

USA

**Fernanda Mariano**

Brazil

**Elismauro Mendonca**

Brazil

**Ranko Mladina**

Croatia

**Markus Moessner**

Germany

**Tsutomu Nakashima**

Japan

**George Noussios**

Greece

**Toyoaki Ohbuchi**

Japan

**Bolajoko O. Olusanya**

Nigeria

**Fredrik Petersson**

Singapore

**Antonio Schindler**

Italy

**Aiden Shearer**

USA

**Sivakumaran Theru Arumugam**

USA

**Valentino Valentini**

Italy

**Fabiana Valera**

Brazil

**Katrien Vermeire**

Belgium

**Petros Vlastarakos**

UK

**Che-Ming Wu**

Taiwan

**Hasan Yasan**

Turkey

**Bülent Yazici**

Turkey

